# Prevalence of potentially inappropriate medications use among older adults and risk factors using the 2015 American Geriatrics Society Beers criteria

**DOI:** 10.1186/s12877-019-1168-1

**Published:** 2019-05-29

**Authors:** Tariq M. Alhawassi, Wafa Alatawi, Monira Alwhaibi

**Affiliations:** 1 Department of Clinical Pharmacy, College of Pharmacy, King Saud University, Riyadh, 11149 Saudi Arabia; 2 Medication Safety Research Chair, College of Pharmacy, King Saud University, Riyadh, Saudi Arabia; 3 Clinical Pharmacy Department, Faculty of Pharmacy, University of Tabuk, Tabuk, Saudi Arabia

**Keywords:** Elderly, Ageing, Beers criteria, Inappropriate prescribing

## Abstract

**Background:**

Older patients are commonly prescribed multiple medications therefore; medication misadventures are common and expected among older patients. The use of potentially inappropriate medicines (PIMs) further contributes to this risk. Therefore, this study aimed to examine PIMs use among older patients using the 2015 Beers criteria.

**Methods:**

A cross-sectional retrospective study using electronic medical records data from a large tertiary hospital in Saudi Arabia was conducted. Older adult patient’s (age ≥ 65 years) who were treated in the ambulatory care setting were included. PIMs use was defined using the 2015 Beers criteria. Descriptive statistics and logistic regression were used to describe and identify potential predictors of PIMs use. All statistical analyses were carried out using the Statistical Analysis Software version 9.2 (SAS® 9.2).

**Results:**

This study included 4073 older adults with a mean age of 72.6 (± 6.2) years. The majority of the study population was female (56.8%). The Prevalence of PIMs to be avoided among older adults was 57.6% where 39.9% of the older adults population were prescribed one PIMs, 14.5% two PIMs, and 3.3% were on three or more PIMs. The most commonly prescribed PIMs were gastrointestinal agents (35.6%) and endocrine agents (34.3%). The prevalence of PIMs to be used with caution was 37.5%. Polypharmacy and existence of certain chronic comorbidities were associated with high risk of PIMs use among older patients.

**Conclusions:**

Given high prevalence of PIMs occurrence among this population, future research on strategies and interventions rationing PIMs use in the geriatric population are warranted.

## Background

The United Nation estimated that the population of older adults defined as those age 65 or older will almost double in Saudi Arabia from 3% in 2000 to 6% or more by the year 2025 [1]. As older adults population is growing, the prevalence of chronic comorbid health conditions secondary to the inevitable nature of ageing expected to increase. This, therefore, is potentially associated with an increase in the use of multiple drugs (polypharmacy) to well manage these comorbidities or to prevent associated complications [2].

Polypharmacy among older adults is common and consequently older patients are at higher risk of potentially inappropriate medications (PIMs) use [3]. PIMs are defined as “medications that should be avoided due to their risk which outweighs their benefit and when there are equally or more effective but lower risk alternatives are available” [4]. PIMs are considered one of the commonly encountered medication-related problems among the older population. The use of PIMs is commonly evaluated using different scales and criteria such as the Beers criteria, which are a set of explicit criteria to identify PIMs. It was first developed in 1991 and consequently updated with the latest update in 2015 [5].

It is well known that PIMs use among older patients is associated with negative health consequences and can impact patients’ quality of life. PIMs use increases the risk of hospitalization, drug-related problems and other adverse health outcomes by two to three folds [6, 7]. For example, drug-related problems secondary to the inappropriate use of sedative and hypnotic among older adults are found highly associated with risk of falls, delirium, and hallucination [8, 9]. Moreover, PIMs use is also associated with an increased cost burden on healthcare system which requires further research to rationalize the use of such medications [10].

The estimated prevalence of PIMs among older patients is high and more than one-third of the older population found to be prescribed at least one PIMs or been exposed to a PIM [11–13]. In the Middle East, the prevalence of PIMs is very high where two studies conducted in Qatar and Lebanon found that 38.3 and 45.2% of older patients were prescribed PIMs respectively [14, 15]. In Saudi Arabia, the prevalence of PIMs use among older adults was assessed by two studies. The first study had identified the PIMs that should be avoided in older patients using 2003 Beers criteria [16]. This study reported that 43% of the older adults used at least one PIM, 18% have used two PIMs and 38.4% have used three or more PIMs. The second study was carried among older patients who visited family medicine clinics and patients who received home health care program [17]. This study found that more than half of the study cohort used one or more of PIMs and majority of these inappropriate medications were avoidable.

Factors associated with inappropriate medications use are variable. Females, older age, polypharmacy, having multiple prescribers physicians, and having poor health status are more likely to be associated with PIMs use [18, 19]. Moreover, certain chronic conditions such as diabetes, hypertension, depression, osteoporosis, and dementia have also been associated with a higher risk of PIMs use compared with older adults who don’t have these chronic conditions [14, 15].

Although many studies have examined the PIMs use among older adults using Beers criteria globally, still few studies has examined factors associated with PIMs use among older adults in Saudi Arabia using the American Geriatric Society (AGS) Beers criteria. While one study was conducted in a Military hospital [16] and another was limited to family medicine [17] ward, both studies were purely descriptive and have used 2003 Beers Criteria. The unique of his study is that it has included all patients admitted to a large tertiary hospital and covers all elderly from all the hospital wards using the electronic medical records which allowed us to get a large sample size to do both descriptive and inferential analysis. Therefore, the main objectives of this study are to assess the prevalence of PIMs use among older adults’ patients in the ambulatory care setting, and to explore factors associated with increased risk of PIMs use among this population.

## Methods

### Study design

A cross-sectional retrospective study conducted using 12-month (1st Jan 2016 to 30th Dec 2016) data extracted from the Electronic Health Record (EHR) database.

### Study population and setting

This study had included older patients aged 65 years and older who visited the ambulatory care clinics in a tertiary teaching hospital in Riyadh, Saudi Arabia.

### Data source and data extraction

This study had used 12-month data retrieved from the EHR database. The study was approved by the Institutional Review Board (IRB) of King Saud University (reference number E-17-2580). All the participants provided written informed consent. Data collected included patient’s demographic profile, clinical data, and medication related data. The demographics file contained information about patients’ date of birth, gender, marital status, nationality. Clinical data provides information about documented medical diagnoses. Physicians reported clinical diagnosis using the International Classifications of Diseases – 9th edition, Clinical Modification (ICD-9-CM) codes or the Systematized Nomenclature of Medicine (SNOMED) diagnosis codes. (Appendix). Medication data contained information about patients prescribed medications as ambulatory care patients such as medication group, date of dispensing, and quantity dispensed.

### Data handling

After obtaining the approval from the study site IRB, data were extracted by trained health informatics pharmacists. The confidentiality of the data used was maintained throughout the research process. Data extracted on Microsoft excel file was stored on a secure, password-protected, and limited accessed computer. Patients’ records were coded to protect patient confidentiality.

### Measures

### Dependent variable: *Potentially Inappropriate Medications (PIMs)*

The main outcome of interest in this study was to estimate the prevalence of PIMs in older adults. The PIMs were identified according to American Geriatric Society (AGS) 2015 updated Beers criteria by applying two categories: (1) medications to avoid for most older adults, and (2) medications to be used with caution [5]. The prevalence of PIMs use was classified into (one PIM, two PIMs, and three or more PIMs). Then PIMs use was classified into two categories: 1) PIMs use (i.e., use of one or more PIMs) and 2) Non-PIMs use (i.e., no PIMs use).

### Independent variables

Independent variables included demographics (age in years, gender, nationality “Saudi, non-Saudi” and marital status “married, unmarried”). Independent variables also included chronic conditions which were categorized into: cardiovascular diseases (hypertension, diabetes, dyslipidemia, heart failure (HF), ischemic heart disease (IHD)); respiratory diseases (asthma and chronic obstructive pulmonary disease (COPD)); musculoskeletal diseases (osteoarthritis and osteoporosis); mental health conditions (depression, anxiety and dementia); chronic kidney disease (CKD) and cancer. Polypharmacy use defined as the use of five medications and more was included.

### Statistical analysis

Data were entered into a custom-designed Microsoft Excel database and analyzed using the Statistical Analysis Software version 9.2 (SAS® 9.2). Descriptive statistics were used to describe the study population. Descriptive statistics were expressed as the mean and standard deviation (±SD) for continuous variables and frequencies and percentages for categorical variables. Bivariate analyses using the Student’s t-test, Pearson’s chi-squared test were used to assess the difference in demographics and disease characteristics between patients with and without PIMs. A two-tailed probability value of < 0.05 was considered to be statistically significant for all analyses. Logistic regression was used to examine the associations between PIMs use and the patient’s age, gender, polypharmacy and different chronic conditions. All statistical tests were performed at a significance level of α = 0.05 and a 95% confidence interval (CI).

## Results

### Description of the study population

In this study, 4073 older adults (age ≥ 65 year) who visited ambulatory care clinics in a tertiary hospital during a 1 year period were identified and included. The mean age was (72.6 ± 6.2) years and the majority of the study population were females. The majority of the study population had two or more chronic conditions (77.9%) and 80.5% were using polypharmacy. Characteristics of the study population are presented in (Table 1).

**Table 1:** Characteristics of the Study Population^a^

Characteristics	*N*	%
Total	4073	100.0
Age mean (±SD)	72.6 (±6.2)	
Marital Status		
Single	157	4.3
Married	3488	95.7
Gender		
Male	1759	43.2
Female	2314	56.8
Nationality		
Saudi	3737	91.9
Non-Saudi	331	8.1
Comorbidities Hypertension		
Yes	3007	73.8
Diabetes		
Yes	2309	56.7
Dyslipidemia		
Yes	2209	54.2
Heart failure		
Yes	51	1.3
Ischemic Heart disease		
Yes	254	6.2
Chronic kidney disease		
Yes	119	2.9
Cancer		
Yes	123	3.0
Asthma		
Yes	403	9.9
COPD		
Yes	17	0.4
Osteoarthritis		
Yes	373	9.2
Osteoporosis		
Yes	344	8.4
Anxiety		
Yes	376	9.2
Depression		
Yes	60	1.5
Dementia		
Yes	25	0.6
No. of chronic conditions		
No chronic	234	5.7
one chronic condition	665	16.3
≥ two chronic conditions	3174	77.9
Polypharmacy		
0 to 4 drugs	794	19.5
≥ 5	3279	80.5

^a^Note: Study Population Comprised of 4073 (age ≥ 65 year) who visited ambulatory care clinics from tertiary hospital

### Prevalence of PIMs

The prevalence of PIMs to be avoided among older adults was (57.6%) (Table 2, Table 3). The most commonly prescribed PIMs to be avoided for older adults were gastrointestinal and endocrine agents. The prevalence of PIMs to be used with caution was 37.5%. The most commonly prescribed PIMs to be used with caution were diuretics followed by antidepressants.

**Table 2:** Prevalence of Potentially Inappropriate Medications Using Updated Peers Criteria among Older Patients^a^

	Number	Percent
PIMs “That Should Be Avoided”		
Yes	2346	57.5
No	1727	42.4
Numbers of PIMs Use That Should Be Avoided		
One PIM	1625	39.9
Two PIMs	588	14.4
Three or more PIMs	133	3.3
PIMs “That Should Be Used With Caution”		
Yes	1529	37.5
NO	2544	62.5
Numbers of PIMs Use With Caution		
One PIM with caution	1341	32.9
Two PIMs with caution	174	4.3
Three or more PIMs with caution	14	0.3

^a^Note: Study population comprised of 4187 (age > 65 year) who visited ambulatory care Clinics from tertiary hospital

**Table 3:** Potentially Inappropriate Medications to Be Avoided For Most Older Adults According to Medication Groups

Medication Groups	*N*	(%)
Gastrointestinal Agents	1450	35.6
Endocrine Agents	1397	34.3
NSAID Agents	278	6.8
Antidepressant Agents	19	0.5
Antispasmodic Agents	20	0.5
Antipsychotic Agents	8	0.2
Anti-infective Agents	7	0.2
Genitourinary medications	4	0.1
Central Alpha Blocker Agents	1	0.02
Peripheral Alpha-1 Blocker Agents	1	0.02
Potentially Inappropriate Medications to Be Used With Caution in Older Adults		
Diuretics	1354	33.2
Antidepressant SSRI	200	4.9
Anticoagulant Agents	54	1.3
Vasodilators	54	1.3
Anticonvulsant Agents	27	0.6
Antidepressant Alpha-2 Antagonist Agents	19	0.5
Anti-neoplastic alkylating Agents	13	0.3
Anti-neoplastic Agent, Antimicrotubule	9	0.2

### Factors associated with PIMs use on bivariate analysis

On bivariate analysis older adults who had chronic conditions compared to those without chronic conditions including hypertension, diabetes, dyslipidemia, HF, IHD, CKD, cancer, COPD, osteoarthritis, osteoporosis and anxiety were all associated with PIMs use. For example, the rate of PIMs use was higher among older adults with hypertension (59.9%, *P*-value < 0.001) as compared to those without hypertension, PIMs use was higher among patients with diabetes (66%, *P*-value < 0.001) as compared to those without diabetes. Moreover, PIMs use was higher among older patients with polypharmacy (66.7%, *P*- value < 0.001) as compared to those without polypharmacy use. Other factors were not associated with PIMs use among older patients (Table 4).

**Table 4:** Number and Raw Percentage of Characteristics by PIM Use* Adjusted Odds Ratios and 95% Confidence Intervals From Logistic Regression on PIM Use among Older Patients

	PIM Use		No PIM Use				PIM Use		
	*N*	%	*N*	%	*P* value	Sig.	OR	95% CI	Sig.
Total	2346	57.5	1727	42.4					
Age Mean	72.8		72.3		0.23				
Marital Status					0.77				
Single	88	56.1	69	43.9					
Married	1995	57.2	1493	42.8					
Gender					0.36				
Male	999	56.8	760	43.2					
Female	1347	58.2	967	41.8					
Nationality					0.25				
Saudi	2165	57.9	1572	42.1					
Non-Saudi	181	54.7	150	45.3					
Hypertension					0.00	***			
Yes	1802	59.9	1205	40.1			0.95	[0.79, 1.13]	
Diabetes					0.00	***			***
Yes	1523	66.0	786	34.0			2.03	[1.76, 2.34]	
Dyslipidemia					0.00	**			
Yes	1320	59.8	889	40.2			0.98	[0.84, 1.13]	
Heart failure					0.00	***			***
Yes	48	94.1	13	5.9			8.19	[2.36, 28.38]	
Ischemic Heart disease					0.00	***			***
Yes	207	81.5	47	18.5			2.74	[1.93, 3.88]	
Chronic kidney disease					0.00	***			***
Yes	97	81.5	22	18.5			2.34	[1.41, 3.87]	
Cancer					0.00	***			***
Yes	97	78.9	26	21.1			2.71	[1.65, 4.44]	
Asthma					0.46				
Yes	239	59.3	164	40.7					
COPD					0.03	*			
Yes	14	82.4	31	17.6			0.72	[0.17, 3.01]	
Osteoarthritis					0.00	**			***
Yes	191	51.2	182	48.8			0.61	[0.48, 0.76]	
Osteoporosis					0.00	**			***
Yes	172	50.0	172	50.0			0.63	[0.49, 0.79]	
Anxiety					0.00	***			**
Yes	262	69.7	114	30.3			1.5	[1.15, 1.96]	
Depression					0.68				
Yes	33	55.0	27	45.0					
Dementia					0.51				
Yes	16	64.0	19	36.0					
Polypharmacy					0.00	***			***
> =5	2188	66.7	1091	33.3			7.79	[6.36, 9.54]	
0 to 4 drugs	158	19.9	636	80.1					

Note: Study population comprised of 4187 (age > 65 year) who visited ambulatory care from tertiary hospital

The reference category for all chronic conditions was “No”

T-test was used to assess the association between age and PIM use

*CI* Confidence Interval, *OR* Odds Ratio, *Ref* Reference Group

Asterisks (*) represent significant differences in polypharmacy

***P < .001; **.001 ≤ *p* < .01; *.01 ≤ *p* < .05

### Factors associated with PIMs use in regression analysis

All factors associated with PIMs use in the bivariate analysis were included in the regression analysis. PIMs use was more likely among older adults with diabetes, HF, IHD, CKD, cancer, osteoarthritis, osteoporosis, and anxiety. The adjusted odds ratios (AOR) and 95% confidence intervals (CI) for factors associated with PIMs use are displayed in (Table 4). Older patients with polypharmacy use were seven folds more likely to have PIMs use compared to older adults with no polypharmacy use (Table 4).

## Discussion

This study aimed to estimate the prevalence of PIMs use among older patients using the latest updated of Beers criteria “the 2015 American Geriatrics Society Criteria”. The prevalence was assessed by using two categories of 2015 Beers criteria; the prevalence of PIMs to be avoided for older adults which was 57.6%, and the prevalence of PIMs that’s should be used with caution was 37.5%. The prevalence of PIMs was relatively high; however, this rate is within the range comparable to the results of previous studies where the prevalence of PIMs ranged between 21 to 58% [20–23]. This variation between studies may be due to using a different setting, study design or different version of Beers criteria. For instance, a study showed a difference in the prevalence of PIMs when they used two versions of beers criteria 2003 and 2012 on the same population (48% versus 59% respectively) [24].

The most likely factor associated with PIMs use in this study was polypharmacy. We found that 80% of this study population used more than five medications. The higher rate of polypharmacy use in our study population can be attributed to the higher rate of multiple chronic conditions (i.e., two or more chronic conditions), in which they may need to take many medications to control their chronic conditions or to prevent complications associated with certain chronic conditions. Several studies have reported an increased risk of PIMs with polypharmacy where one study showed that PIMs use was two times higher among older patients with polypharmacy, while another study reported that PIMs use was three times as likely with polypharmacy use [25, 26, 21, 22].

In this study, the presence of certain chronic conditions in older patients predicted the increased chance of PIMs use including diabetes, IHD, HF, CKD, cancer, osteoarthritis, osteoporosis, and anxiety. Multiple studies have demonstrated a significant association between PIMs use and cardiovascular diseases, diabetes, osteoporosis and increase number of chronic diseases [27]. The association between PIMs use and different predictors such as the presence of certain chronic conditions and polypharmacy use, although this is not a novel finding, however, this could be an indicator of inappropriate medication management for these conditions in such vulnerable population [28, 29]. This finding can also help to understand the factors associated with PIMs use, as having this knowledge makes it possible to assess health care provided to the older population and the prompt need for future services directed towards older patients. The role of health care providers should expand in order to take the necessary precautions when managing older patient’s conditions to avoid inappropriate medications prescribing, adverse events and other misadventures associated with older patients. Additionally, pharmacists can play a major role in improving the appropriateness of medications use by the recommendation for either medication discontinuation, medication review, the clinical application of tools to assess PIMs such as Peers criteria, or other tools to identify older patients at risk of unnecessary use of PIMs [30].

The study has some limitations. Firstly, this study did not apply other categories of 2015 Beers criteria and only PIMs to be avoided and PIMs to be used with caution were included in the study and this was due to the nature of this study design as a retrospective study and required patient’s data to identify other category of PIMs was not recorded in EHR database. Secondly, findings of this study cannot be generalized to all older adults across Saudi Arabia or the older population entirely as this study included only older patients who visited ambulatory care clinics of one tertiary hospital. Thirdly, the impact of other factors such as the socio-demographic predictors, variation between clinical settings in the region, comorbidity index or recent hospitalization were not evaluated in this study requiring future studies to comprehensively assess such factors and PIM use among older patients. Further, we were not able to capture the use and failure of other drugs prior to the prescribing of PIMS given the nature of the study design.

However, this study still can be considered novel as the study was designed using the latest Beers criteria which also considered one of the most utilized criteria for identifying PIMs among older adults in clinical setting and the latest two update of Beers criteria were supported by American Geriatric Society which improved the quality of the criteria by application of an evidence-based approach [31]. Moreover, to our knowledge, this is the first study to explore PIMs use among older patients and evaluate predicating factors associated with PIMs use in Saudi Arabia. Therefore, this study shed light more on what is needed to understand how to reduce harms associated with the unnecessary use of PIMs and provide better health care for older adults to minimize medications risk and economic burden. Consequently, findings of this study address important information to policymakers about a serious need for effective implantation of pharmacy services such as medication therapy management and continuous medication review regularly to reduce the use of PIMs. Also, elderly patients may benefit from a multidisciplinary collaborative care model that involves pharmacist follow up for the patients to assess the medication use and minimize inappropriate medications. Furthermore, the policymaker would benefit from conducting continuous educational activities for healthcare providers to help them understand the guidelines and criteria on proper prescribing of medications for the elderly population.

## Conclusions

This study showed a high prevalence of PIMs that should be avoided or used with caution among older patients. Polypharmacy and chronic conditions were predictors for increased use of PIMs among older patients. With the anticipated growth of the older population, future studies to explore the adverse health outcome associated with PIMs use and strategies to rationalize the use of unnecessary or high-risk medications among this population are warranted.

## Data Availability

The EHR dataset used during and/or analyzed during the current study are not publicly available due to our IRB policy but are available from the corresponding author upon reasonable request.

## Appendix

**Table 5:** International Classifications of Diseases – 9th edition, Clinical Modification (ICD-9-CM) codes or the Systematized Nomenclature of Medicine (SNOMED) diagnosis codes

Type of Chronic Conditions	ICD-9-CM Codes	SNOMED Codes
Cardiovascular Conditions		
Hypertension	401.9	64,176,011, 2,164,904,016
Diabetes	250, 250.00	121,589,010, 502,372,015
Ischemic heart disease		2,534,671,011, 2,537,479,013, 397,667,016, 2,534,663,012
Vascular heart disease		1,705,016
Stroke		2,644,233,012, 2,476,091,017, 345,636,015, 345,682,011
Heart failure	428.0, 428.1	1,234,906,013, 143,156,018, 251,680,018, 94,251,011, 2,645,367,010, 18,472,010, 139,475,013, 2,816,764,017, 493,289,014, 70,653,017, 80,720,010
Dyslipidemia		92,826,017, 1,209,706,018
Musculoskeletal Conditions		
osteoarthritis		1,776,248,011, 359,420,013, 359,421,012, 1,785,522,017
Osteoporosis		453,855,011, 107,806,013
Respiratory Conditions		
Asthma	493, 493.90	301,485,011, 301,480,018
COPD		23,290,013, 23,287,019, 475,431,013, 475,427,019
Mental Health Conditions		
Dementia		87,274,019
Depression	311	486,186,018, 486,187,010, 110,183,011, 346,973,011, 346,979,010, 55,208,011, 454,082,014, 486,187,010, 124,707,013, 1,208,903,011, 490,537,016, 346,980,013, 1,228,731,019, 486,184,015
Anxiety		346,980,013, 369,987,018, 303,689,015, 481,155,011, 81,133,019
Other chronic diseases		
Chronic kidney disease		2,771,041,011, 2,767,385,013, 150,315,015
Cancer^a^	153.9, 202.80,202.8	1,217,470,011, 379,663,018, 379,662,011, 1,786,810,016, 1,228,536,014, 1,228,535,013, 1,228,484,019, 157,732,017, 1,783,096,018, 198,367,013, 1,220,412,013, 198,006,010, 414,270,011, 2,663,377,018, 414,271,010, 675,125,016, 195,620,018, 1,783,096,018, 413,121,012, 1,778,963,014, 2,663,475,013, 1,220,409,010, 1,228,486,017, 1,228,547,019, 1,479,600,014, 1,786,665,019, 1,229,105,017

^a^Cancer included anal, brain, breast, bladder, colon, endometrial, esophageal, gastric, leukemia, liver, lung, lymphoma, ovary, rectal, thyroid, pancreatic, prostate, and uterus cancer

## Abbreviations

AGS: American Geriatric Society
AOR: Adjusted Odds Ratios
CI: Confidence Intervals
CKD: Chronic Kidney Disease
COPD: Chronic Obstructive Pulmonary Disease
EHR: Electronic Health Record
HF: Heart Failure
IHD: Ischemic Heart Disease
IRB: Institutional Review Board
PIMs: Potentially Inappropriate Medicines
